# Pan-cancer analysis of DDIT4 identifying its prognostic value and function in acute myeloid leukemia

**DOI:** 10.1007/s00432-024-05676-8

**Published:** 2024-03-20

**Authors:** Fangmei Li, Jiyu Miao, Rui Liu, Ru Zhang, Aili He

**Affiliations:** 1 Department of Hematology, The Second Affiliated Hospital of Xi’an Jiaotong University, NO.157 Xiwu Road, Lianhu District, Xi’an City, 710004 China; 2 Xi’an Key Laboratory of Diagnosis and Treatment of Hematological Diseases, Xi’an, China

**Keywords:** Acute myeloid leukemia, DNA-damage-inducible transcript 4, Diagnosis

## Abstract

**Background:**

Acute myeloid leukemia (AML) is a hematological malignancy derived from the accumulation of abnormal proliferation of infantile leukocytes in the hematopoietic system. DNA-damage-inducible transcript 4 (*DDIT4*) acting as a negative regulator of rapamycin inhibitor is involved in various cellular functions. Many studies have suggested that *DDIT4* plays a key role in tumorigenesis. However, the role of *DDIT4* in AML has been poorly studied.

**Method:**

In this study, we analyzed the expression of *DDIT4* in AML patients using The Cancer Genome Atlas and real-time polymerase chain reaction. The Chi-square test was used to assess the correlation between *DDIT4* and clinical characters in AML patients. Loss-of-function experiments were implemented to investigate the role of *DDIT4* in AML carcinogenesis. The R package was applied to evaluate the correlation between *DDIT4* expression and immune cells.

**Results:**

Results showed that the expression of *DDIT4* was associated with Age, Cytogenetic risk, Cytogenetics and OS event. Moreover, high expression of *DDIT4* led to a terrible prognosis. KEGG analysis showed that differently expressed genes (DEGs) were involved in the PI3-Akt signaling pathway. GSEA enrichment analysis displayed DEGs were correlated with apoptosis. Functional experiments presented that knocking down *DDIT4* suppressed cell cycle transition/proliferation and facilitated apoptosis. In addition, *DDIT4* is associated with immune infiltration.

**Conclusion:**

Our research verified that *DDIT4* can be used as a prognostic marker and a potential therapeutic target for AML.

## Introduction

The incidence of AML is approximately 4.1 per 100,000 women and men per year. The mortality rate of AML is 2.7 per 100,000 per year (2021). AML is a genetically heterogenous blood tumor, characterized by the uncontrolled clonal overexpansion of undifferentiated myeloid progenitor cells (Döhner et al. 2015). Chemotherapy is the main treatment for AML, although most patients can achieve complete remission with chemotherapy, many patients finally relapse or are resistant to chemotherapy (Reville and Kadia 2020). Improving the ability to predict treatment response, duration of remission, and likelihood of recurrence can optimize the prognosis of AML (Small et al. 2022). The newest AML classification system, published in 2022, incorporates new pathological findings and emphasizes the integration of molecular analysis into the diagnosis of AML (Shimony et al. 2023). As the prognosis of AML remains dismal, there is a strong need for advances in biomarkers to guide prognostic assessment and risk stratification and to guide better treatment strategies.

*DDIT4*, also known as *REDD1*, regulates cell growth by negatively regulating the mammalian target of rapamycin (mTOR), which is widely expressed in tissues and highly induced by hypoxia and various stressors (Shoshani et al. 2002). *DDIT4* is also highly expressed in various cancer tissues, and the prognosis of patients with high *DDIT4* expression is worse than those with low *DDIT4* expression (Liu et al. 2019). Previous studies have shown that high level of *DDIT4* is strongly associated with poorer outcomes in patients with AML (Ley et al. 2013; Zhao et al. 2018). The prognostic value and function of *DDIT4* in AML have not been thoroughly studied. Our goal was to explore the relevance between *DDIT4* expression and clinical characters in AML and its specific prognostic value. We also explored the impact of *DDIT4* on AML cell proliferation, apoptosis and cell cycle.

## Materials and methods

### Data source

The TCGA data corresponding to pan-cancer and the normal tissue data corresponding to GTEx were extracted from UCSC XENA (https://xenabrowser.net/datapages/) by the Toil process (Vivian et al. 2017) unified handling TCGA and GTEx FPKM RNAseq data format, included 7568 GTEx normal controls, 727 TCGA para-carcinoma tissues, and 9807 TCGA tumor tissues. The 150 AML patients’ clinical data was downloaded from the TCGA database (https://portal.gdc.cancer.gov).

### Expression analysis of *DDIT4* in tumor and normal tissues

To analyze the expression of *DDIT4* in different cancers and their normal counterparts, *t*-test or Wilcoxon rank sum test was applied. The data were displayed by the ggplot2 package. The Human Protein Atla (HPA) (Colwill and Gräslund 2011) were used to investigate the intensity of immunohistochemical staining of *DDIT4* in lung adenocarcinoma, renal chromophobe carcinoma and prostate cancer tumor tissues.

### Clinical correlation analysis

To explore the prognostic ability of *DDIT4* and its relationship with clinical features, we conducted survival analysis and ROC analysis. R software (version 4.2.1) was used to analyze the prognostic significance of *DDIT4* in different cancers. Survival package was used to test the proportional risk hypothesis and to fit survival regression. The results were visualized using survminer package and the ggplot2 package. Clinical features were analyzed by univariate and multivariate Cox regression using forest plot. Nomograph was established to assess the survival of AML patients based on the results of multivariate logistic model. Calibration curve was used to evaluate the accuracy and clarity of Nomograph.

### Differential expressed genes analysis

AML patients in the TCGA database were divided into two groups with *DDIT4*^high^ expression and *DDIT4*^low^ expression according to the median *DDIT4* expression. Limma package was used to analyze the differentially expressed genes in the two groups and draw the volcano map. The filtering condition was that log2 fold change (|log2 FC|) > 1.0 with an adjusted *P*-value < 0.05.

### Functional enrichment analysis and PPI analysis

We used the ClusterProfiler (version 3.14.2) package to complete Gene Ontology (GO) and Kyoto Encyclopedia of genes and Genomes (KEGG) pathway enrichment analyses on that are differently expressed with *DDIT4* in AML. Gene Set Enrichment Analysis (GSEA) is a computing method that estimates whether an a priori defined set of genes shows statistically significant, consistency between two states. GSEA database (www.broadinstitute.org/gsea) was used to analyze screened differently expressed genes and identify enrichment signaling pathways associated with AML. *P*-value < 0.05 was considered statistically significant.

The STRING database (https://string-db.org/) was used for PPI network analysis. We investigated the correlation between DEGs with a confidence score than 0.4 to establish the PPI network. The visualization of the PPI network was accomplished with the Cytoscape software plugin MCODE (Shannon et al. 2003). Finally, seven important core genes (*THBS1, HGF, IGF1, IL-6, CCL25, MMP7,* and *IL-10*) were obtained through screening of the PPI-related genes and prognostic genes.

### Immunoinfiltration analysis

Based on the ssGSEA algorithm provided by R package –GSVA [1.46.0] (Hänzelmann et al. 2013), this paper utilizes Immunity article (Bindea et al. 2013) provided markers for 24 immune cells to calculate immune infiltration. Spearman correlation analysis was performed between principal variables and immune infiltration matrix data in the data, and the analysis results were visualized with the lollipop chart in ggplot2 package. Spearman correlation analysis was used to analyze the relationship between *DDIT4* and 6 classic immune checkpoint molecules (*TIGIT, PDCD1, LAG3, CD274, HAVCR2* and *CTLA4*), and the analysis results were visualized by ggplot2 package coexpression heat map.

### Cell culture and small interfering RNA transfection

Human leukemia cell lines THP1 and NB4 were obtained from the Cell Bank of Chinese Academy of Science and cultured in RPMI-1640 medium with 10% fetal bovine serum and penicillin–streptomycin at 37 ℃. DDIT4 small interfering RNA and control siRNA were synthesized by GenePharma (Shanghai, China). Transfection was performed using RFECT reagents (Baidai biotechnology, Changzhou, China) according to the manufacturer’s protocol.

### Real-time quantitative polymerase chain reaction (RT-qPCR)

RNA was extracted using Trizol reagent (Sigma, Aldrich), and then reversed into cDNA for RT-qPCR reaction. Primers are as follows: DDIT4, 5′-TGCATTGGGGACACATACCC-3′ and 5′-CCCAAGTGATCCCTGACACC-3′; Actin, 5′-GTGGCCGAGGACTTTGATTG-3′ and 5′-CCTGTAACAACGCATCTCATATT-3′.

### Cell counting kit (CCK) 8 assay

After transfected with DDIT4 siRNA, THP1 and NB4 cells were seeded in 96-well plates, and 10 µl CCK-8 solution (7Sea Pharmatech, China) was added to each well at 0 h, 24 h, 48 h and 72 h, respectively. After incubation for 4 h, absorbance at 450 nm was measured.

### EdU, cell apoptosis and cell cycle assay

After transfection of DDIT4 siRNA, cell proliferation, apoptosis and cell cycle were detected by flow cytometry. Cell proliferation was detected using EdU assay kit (Beyotime, Shanghai, China). Cells were stained with Annexin V-PE/RedNucleus (Beyotime, Shanghai, China) and apoptosis rate was detected. The cells were fixed with cold 70% alcohol for 24 h and then stained with PI solution (Beyotime, Shanghai, China) and Rnase A (Beyotime, Shanghai, China) for cell cycle detection.

### Western blotting

The cells were lysed with RIPA reagent (Xianfeng Biotechnology, Xi’an, China) and quantified by BCA Protein Assay Kit (Beyotime, Shanghai, China). 40 μg of total protein was added to 10% SDS-PAGE and then transferred to 0.2 mm PVDF membrane (Millipore). Incubate 5% skim milk at room temperature for 1 h and then add the corresponding primary antibody (Proteintech, 1:1000 dilution) for overnight incubation at 4 ℃. After TBST cleaning, add the secondary antibody (Proteintech, 1:10,000) and incubate at room temperature for 1 h. Finally, the protein signal was detected by the ECL system.

### Statistical analysis

The results were shown as mean ± SD. GraphPad Prism software 9.0 and SPSS software 18.0 were adopted for data processing. Chi-square test were performed to explore the correlation between *DDIT4* and clinical features of categorical variables. Student *t*-test or ANOVA test were used for continuous variables. Furthermore, Welch *t*’test, Kruskal–Wallis test and Mann–Whitney *U* test were applied for comparison between two groups. *P* value < 0.05 was considered statistically significant.

## Results

### Pan-cancer analysis of DDIT4

**Fig. 1:**
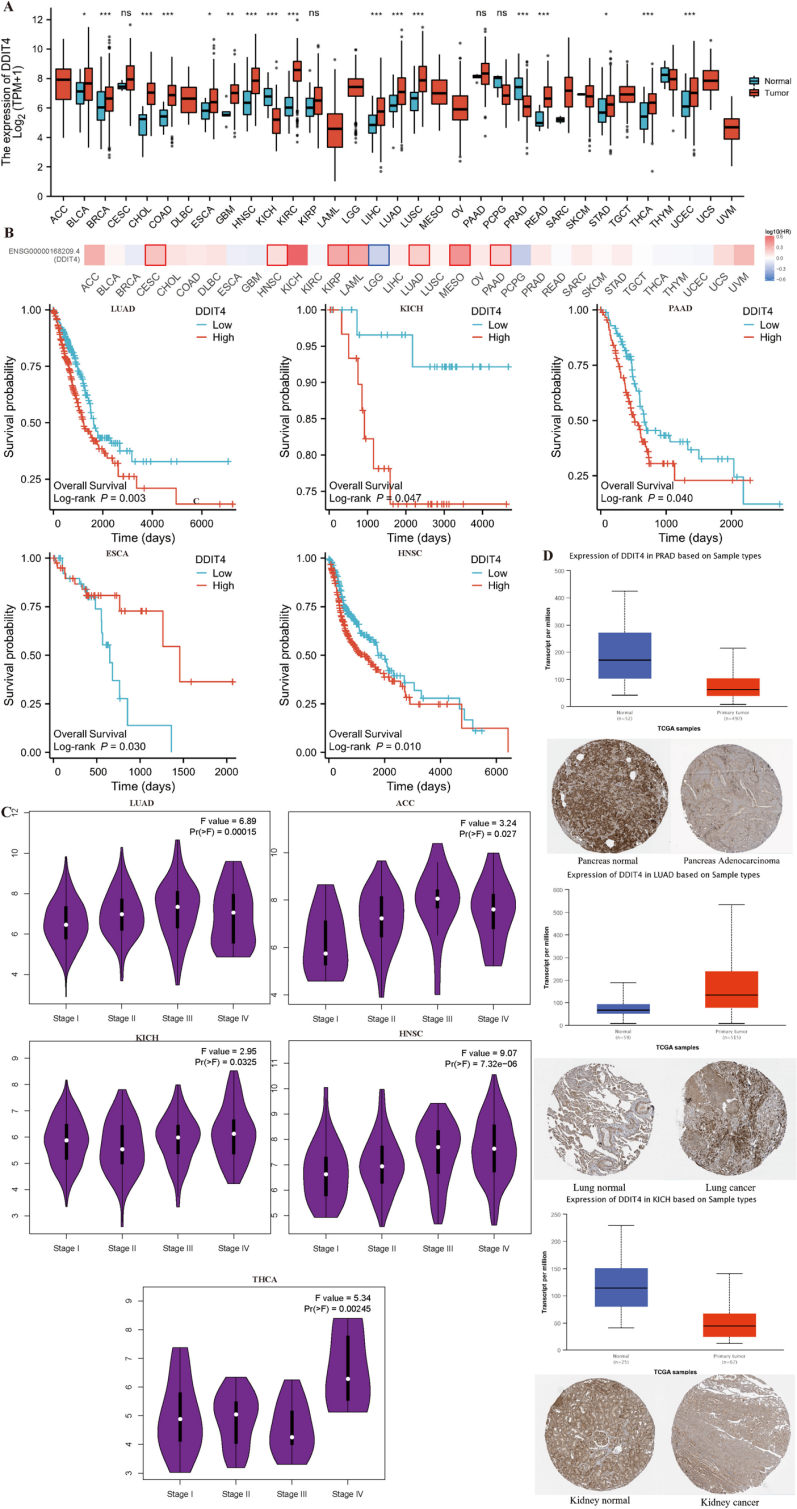
High *DDIT4* expression was associated with adverse clinical outcomes. **A**The expression of *DDIT4* between tumor tissues and normal tissues in TCGA and GTEx databases. **B** The level of *DDIT4* is related to patients’ overall survival. **C** *DDIT4* was expressed differently in different stages of the tumor. **D** Immunohistochemistry detected DDIT4 expression in tumor and normal tissues. (ns *p* ≥ 0.05, *p < 0.05, **p < 0.01, ***p < 0.001)

We analyzed *DDIT4* expression in 33 tumor tissues and corresponding normal tissues in the TCGA and GTEx databases. As shown in Fig. 1A, *DDIT4* was up-regulated in multiple tumors compared with normal controls. As shown in the survival curves in Fig. 1B, we also found that patients with high *DDIT4* expression in LUAD (*p* = 0.003), KICH (*p* = 0.047), PAAD (*p* = 0.040) and HNSC (*p* = 0.010) had worse prognosis. In ESCA (*p* = 0.030), patients with high *DDIT4* expression had a better prognosis than those with low *DDIT4* expression. At the same time, we used GEPIA2.0 website to investigate *DDIT4* expression in different tumor stages. The results showed that *DDIT4* expression was gradually increased with the staged in LUAD, KICH, THCA, ACC and HNSC (Fig. 1C). Immunohistochemical staining results of HPA database showed that *DDIT4* expression decreased in KICH and PARD tumor tissues and increased in LUAD tumor tissues (Fig. 1D), this is consistent with the results of pan-cancer analysis.

### *DDIT4* expression and correlation with clinical features in AML

**Fig. 2:**
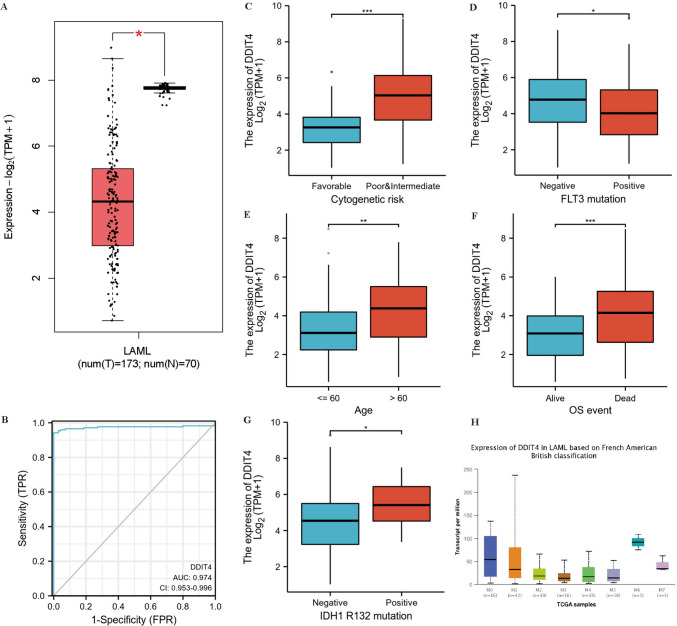
**A** The expression of *DDIT4* was lower than normal control (*n* = 70) in AML (*n* = 173) in the TCGA database. **B** ROC of *DDIT4* in AML. **C**–**G** The expression of *DDIT4* between different genetic risk stratification (**C**), *FLT3* mutation status (**D**), age distribution (**E**), overall survival event (**F**), IDH1 R132 mutation status (**G**) and AML subtypes (**H**). ROC, Receiver operating characteristic analysis (ns *p* ≥ 0.05, *p < 0.05, **p < 0.01, ***p < 0.001)

We compared *DDIT4* mRNA expression levels in AML patients with normal controls in the GEPIA 2.0 database. *DDIT4* expression was downregulated in AML (*p* < 0.05) (Fig. 2A). In addition, the AUC value of the ROC analysis was 0.974 (95% confidence interval, CI 0.953–0.996, Fig. 2B). We then analyzed the relationship between *DDIT4* expression level and clinical features of AML patients. In this study, we analyzed 151 AML patients with clinical information from the TCGA database. As shown in Fig. 2C–G, high *DDIT4* expression was associated with unfavorable cytogenetic risk (*p* < 0.001, Fig. 2C), FLT3 negative mutation (*p* < 0.05, Fig. 2D), older age (*p* < 0.01, Fig. 2E), death (*p* < 0.001, Fig. 2F), and IDH1 R132 positive mutation (*p* < 0.05, Fig. 2G) in AML patients, with no association with French-American-British (FAB) classifications (Fig. 2H). As shown in Table 1, 151 AML patients were further divided into low and high expression of DDIT4 according to the median value of *DDIT4* mRNA expression. Correlation analysis results showed that DDIT4 expression was correlated with Age (*p* < 0.001), Cytogenetic risk (*p* < 0.001), Cytogenetics (*p* = 0.002) and OS event (*p* = 0.001).

**Table 1:** Correlation analysis between *DDIT4* expression level and clinical characteristics in 151 AML patients

Characteristic	*DDIT4* (Low)	*DDIT4* (High)	*p* value
*n*	75	76	
Gender, n (%)			0.122
Female	39 (25.8%)	29 (19.2%)	
Male	36 (23.8%)	47 (31.1%)	
Age, median (IQR)	51 (38.5, 61)	63 (47.75, 71.25)	** < **0.001
WBC count(10^9/L), n (%)			0.874
< = 20	37 (24.7%)	40 (26.7%)	
> 20	37 (24.7%)	36 (24%)	
Cytogenetic risk, n (%)			** < **0.001
Favorable	26 (17.4%)	5 (3.4%)	
Intermediate	37 (24.8%)	45 (30.2%)	
Poor	11 (7.4%)	25 (16.8%)	
FAB classifications, n (%)			0.053
M0	4 (2.7%)	11 (7.3%)	
M1	14 (9.3%)	21 (14%)	
M2	20 (13.3%)	18 (12%)	
M3	11 (7.3%)	4 (2.7%)	
M4	19 (12.7%)	10 (6.7%)	
M5	7 (4.7%)	8 (5.3%)	
M6	0 (0%)	2 (1.3%)	
M7	0 (0%)	1 (0.7%)	
Cytogenetics, n (%)			0.002
Normal	31 (23%)	38 (28.1%)	
+ 8	4 (3%)	4 (3%)	
del(5)	0 (0%)	1 (0.7%)	
del(7)	2 (1.5%)	4 (3%)	
inv(16)	8 (5.9%)	0 (0%)	
t(15;17)	8 (5.9%)	3 (2.2%)	
t(8;21)	7 (5.2%)	0 (0%)	
t(9;11)	0 (0%)	1 (0.7%)	
Complex	7 (5.2%)	17 (12.6%)	
OS event, *n* (%)			0.001
Alive	37 (24.5%)	17 (11.3%)	
Dead	38 (25.2%)	59 (39.1%)	

*n* number of patients, *IQR* Interquartile range, *WBC* White blood cell, *FAB* French–American–British subtype

### *DDIT4* is an independent prognostic factor in patients with AML

**Fig. 3:**
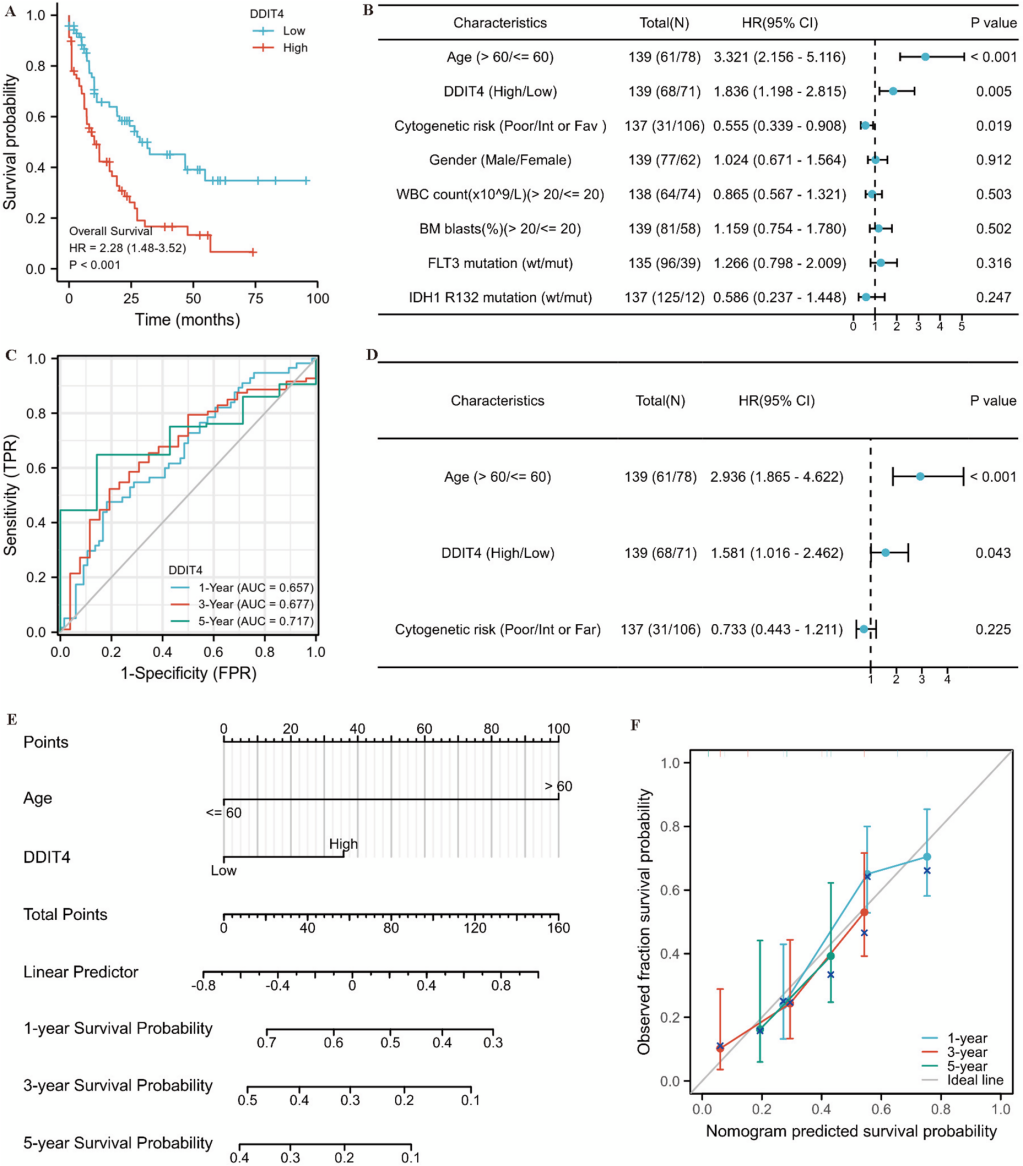
Prognostic value of *DDIT4* expression in AML. **A** DDIT4’s predictive ability on survival for AML, KM curve dispayed OS between high and low *DDIT4* expression groups. **B** Forest plot for univariate analysis results of AML patients. **C** Diagnostic ability of *DDIT4* in AML with 1-,3-, and 5-year. **D** Forest plot for multivariate regression analysis results of AML patients. **E** Nomograph for predicting 1-year, 3-year and 5-year OS in AML patients. **F** Calibration curve of Nomogram. OS overall survival

Kaplan–Meier analysis was used to determine whether the expression level of *DDIT4* was correlated with patient’s prognosis. The results showed that AML patients with high expression of *DDIT4* had shorter overall survival (OS) compared to low expression *DDIT4* cohort (HR = 2.28, *p* < 0.001)(Fig. 3A). To explore the prognostic value of *DDIT4* in AML patients, ROC curves were constructed for 1,3, and 5 year OS. The 1-year, 3-year and 5-year AUCs were 0.657, 0.677, 0.717, respectively (Fig. 3C). Univariate and multivariate regression analyses were used to further explore the predictive value of *DDIT4* for survival. Univariate analysis showed age, cytogenetic risk, and *DDIT4* expression were associated with prognosis (*p* < 0.001,* p* < 0.001, *p* < 0.001, respectively) in AML patients (Fig. 3B). Meanwhile, multivariate regression revealed that age was an independent prognostic factor of poor OS (HR, 2.890; 95%CI, 1.848–4.519; *p* < 0.001) (Fig. 3D). Furthermore, according to these prediction factors, we constructed a nomogram plot including age, cytogenetic risk, and *DDIT4* expression to predict the survival probability at 1,3, and 5 years of AML patients (Fig. 3E). The observation results were consistent with those predicted by nomogram calibration curve (Fig. 3F). In summary, high *DDIT4* expression in AML patients is associated with poor prognosis.

### Differentially expressed genes correlated with *DDIT4* enrichment analysis

**Fig. 4:**
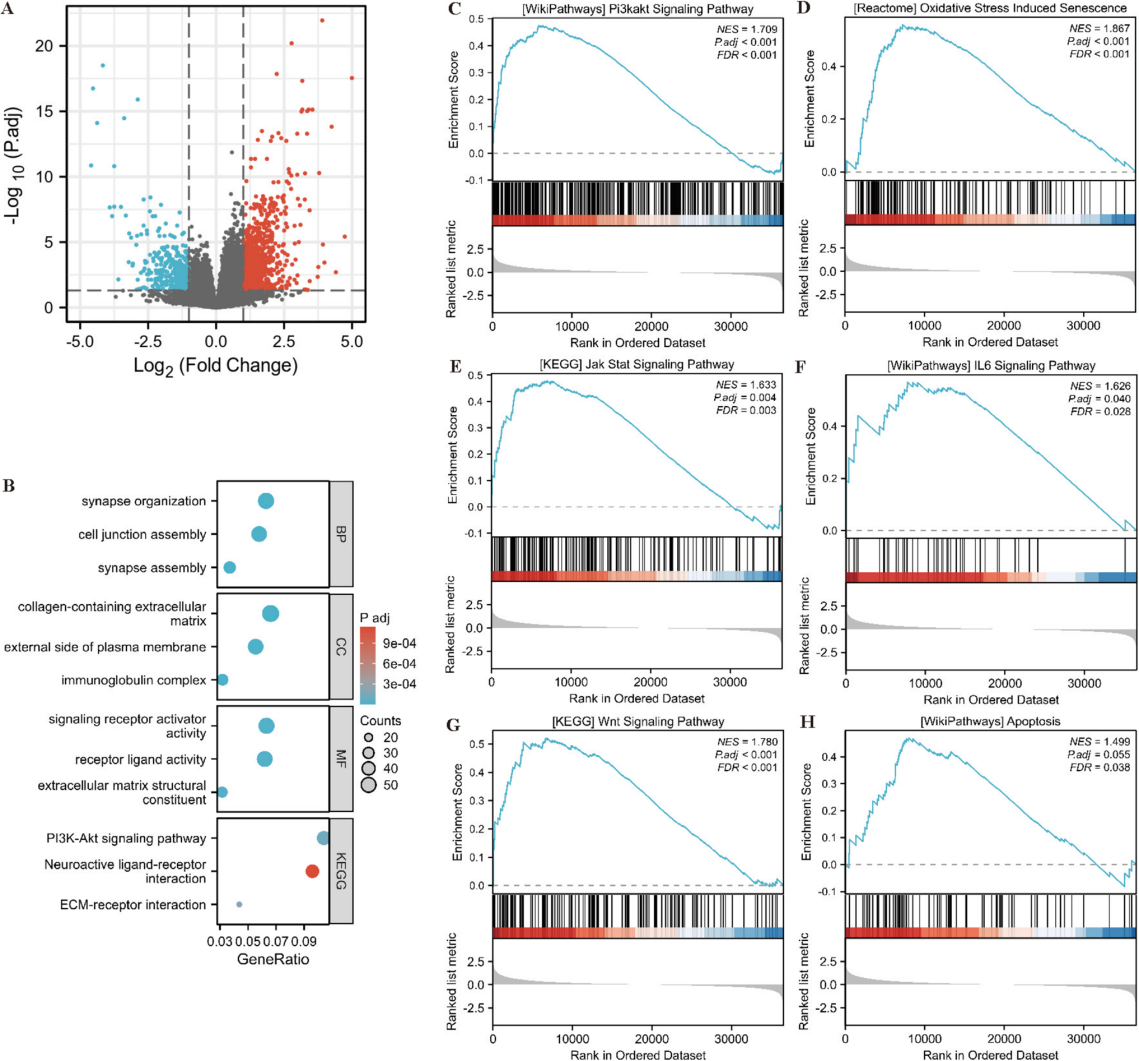
**A** Volcano plot for DEGs among DDIT4^high^ and DDIT4^low^ groups. **B** GO terms and KEGG pathways analysis of DEGs. **C**–**H** GSEA enrichment analysis of DEGs. **C** Enriched genes of Pi3k akt pathways. **D** Enriched genes in Oxidative Stress induced Senecence. **E** Enriched genes of Jak Stat Signaling pathway. **F** Enriched genes of IL6 signaling pathway. **G** Enriched genes of Wnt signaling pathway. **H** Enriched genes in apoptosis pathway. DEGs different expression genes

To further elucidate the mechanism of *DDIT4* in the pathogenesis of AML. We identified genes that were differentially expressed between the high *DDIT4* and low *DDIT4* cohorts of AML patients. A total of 1679 differentially expressed genes (DEGs) were displayed by volcano plot (Fig. 4A), where 1216 genes were up-regulated and 463 genes were down-regulated (|log2FC|> 1, adjusted *p*-value < 0.05). Then we completed GO and KEGG analysis of DEGs using Cluster Profilter software package. KEGG analysis showed that these DEGs were mainly involved in the PI3-Akt signaling pathway, ECM-receptor interaction, Neuroactive ligand-receptor interaction and Neuroactive ligand-receptor interaction (Fig. 4B). Next, GSEA software was used to perform GSEA enrichment analysis on DEGs, and the results showed that *DDIT4* was involved in PI3K-Akt, Jak Stat, IL6, Wnt signaling pathways and Apoptosis, Oxidative Stress Induced Senescence (Fig. 4C–H).

### PPI networks of *DDIT4*-related partners

**Fig. 5:**
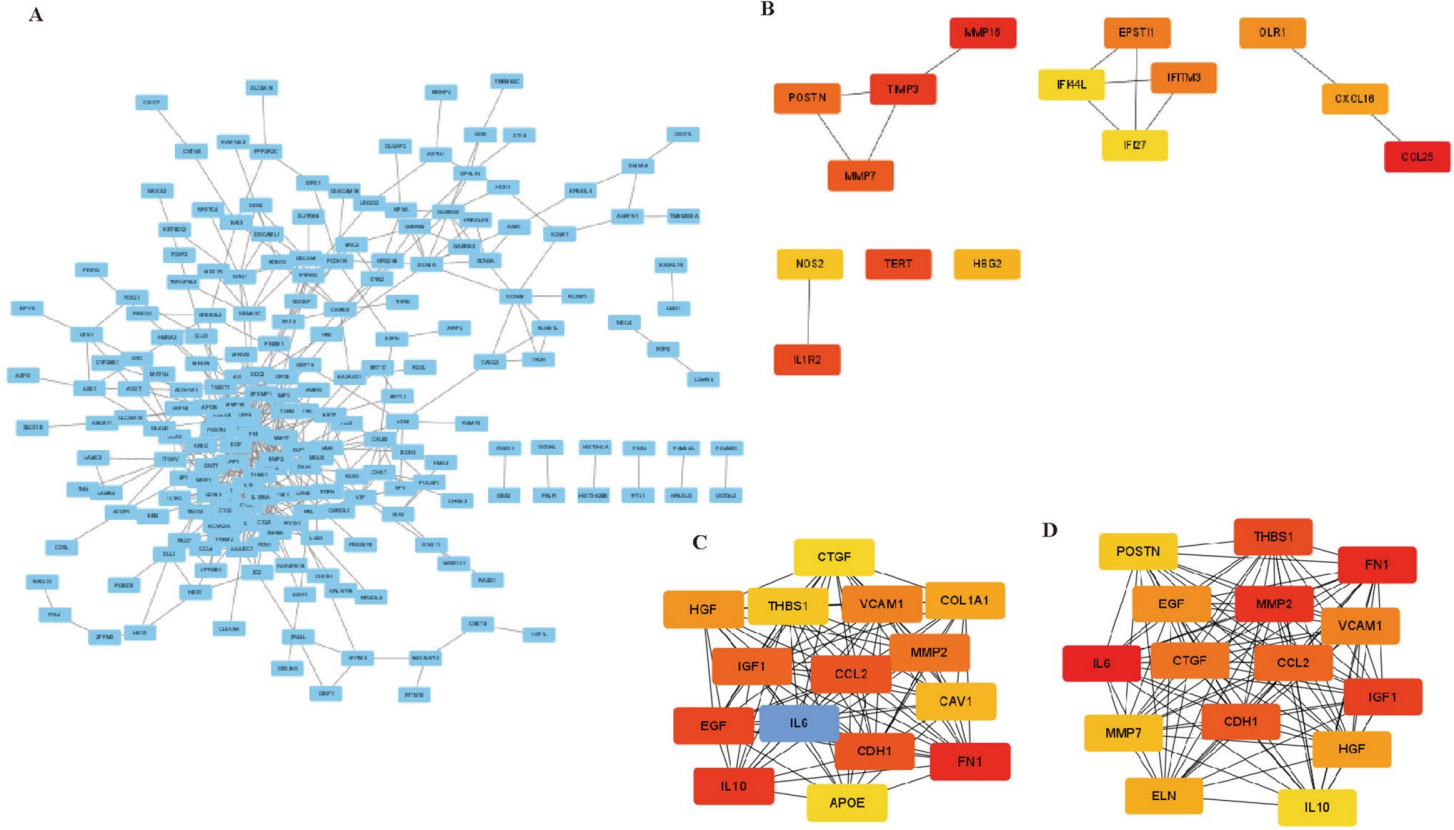
PPI network of 287 encoding DEGs. (**B**–**D**) Hub genes filtrated by DMNC (**B**), MNC(C), and MCC (**D**) analysis. DEGs different expression genes

To identify the hub genes related to DDIT4 in AML, the PPI network of 287 encoding DEGs was constructed using the online bioinformatics tool STRING. The results were visualized using Cytoscape software (Fig. 5A). The PPI network was then analyzed using MCODE, a plugin of Cytoscape software, to search for hub genes. The top 15 hub genes were determined by maximum neighborhood component (DMNC), density of maximum neighborhood component (MNC), and maximal clique centrality (MCC) screening (Fig. 5B–D).

**Fig. 6:**
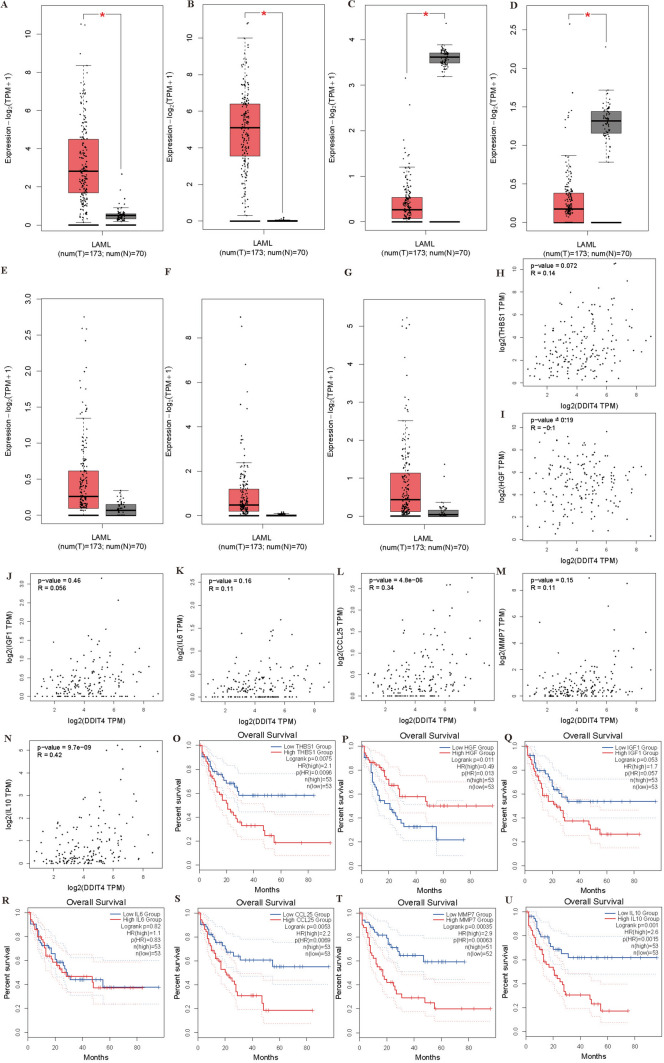
**A**-**G** Expression of 7 core genes in AML patients (*n* = 173) versus healthy controls (*n* = 70). **A** *THBS1*. **B** *HGF*. **C** *IGF1*. **D** *IL-6*. **E** *CCL25*. **F** *MMP7* (**G**) *IL-10*. (**H**–**N**) Correlation analysis between 7 core genes with DDIT4. **H** *THBS1*. **I** *HGF*. **J** *IGF1*. **K** *IL-6*. **L** *CCL25*. **M*** MMP7.* **N** *IL-10*. **O**–**U** OS analysis between high- and low- core genes expression displayed by KM curves. **O** *THBS1*. **P** *HGF*. **Q** *IGF1*. **R** *IL-6*. **S** *CCL25*. **T*** MMP7.* **U** *IL-10*. OS overall survival. (*p < 0.05)

Among these core genes, *THBS1* and *HGF* are highly expressed in AML (Fig. 6A, B), *IGF1* and *IL-6* are low expressed in AML (Fig. 6C, D), and *CCL25*, *MMP7* and *IL-10* are not different from healthy controls (Fig. 6E–G). *CCL25* and *IL-10* were positively correlated with *DDIT4* in AML (Fig. 6L, N), while *THBS1*, *HGF*, *IGF1*, *IL-6*, and *MMP7* were not correlated with *DDIT4* (Fig. 6H–K, M). In addition, patients with high expression of *CCL25*, *THBS1*,*MMP7*, *IGF1* and *IL-10* in AML have worse OS (Fig. 6O, Q, S-U), while patients with HGF high expression has longer OS (Fig. 6P). *IL-6* expression was not correlated with OS in AML patients (Fig. 6R).

### Oncogenic function of *DDIT4* in AML cells

**Fig. 7:**
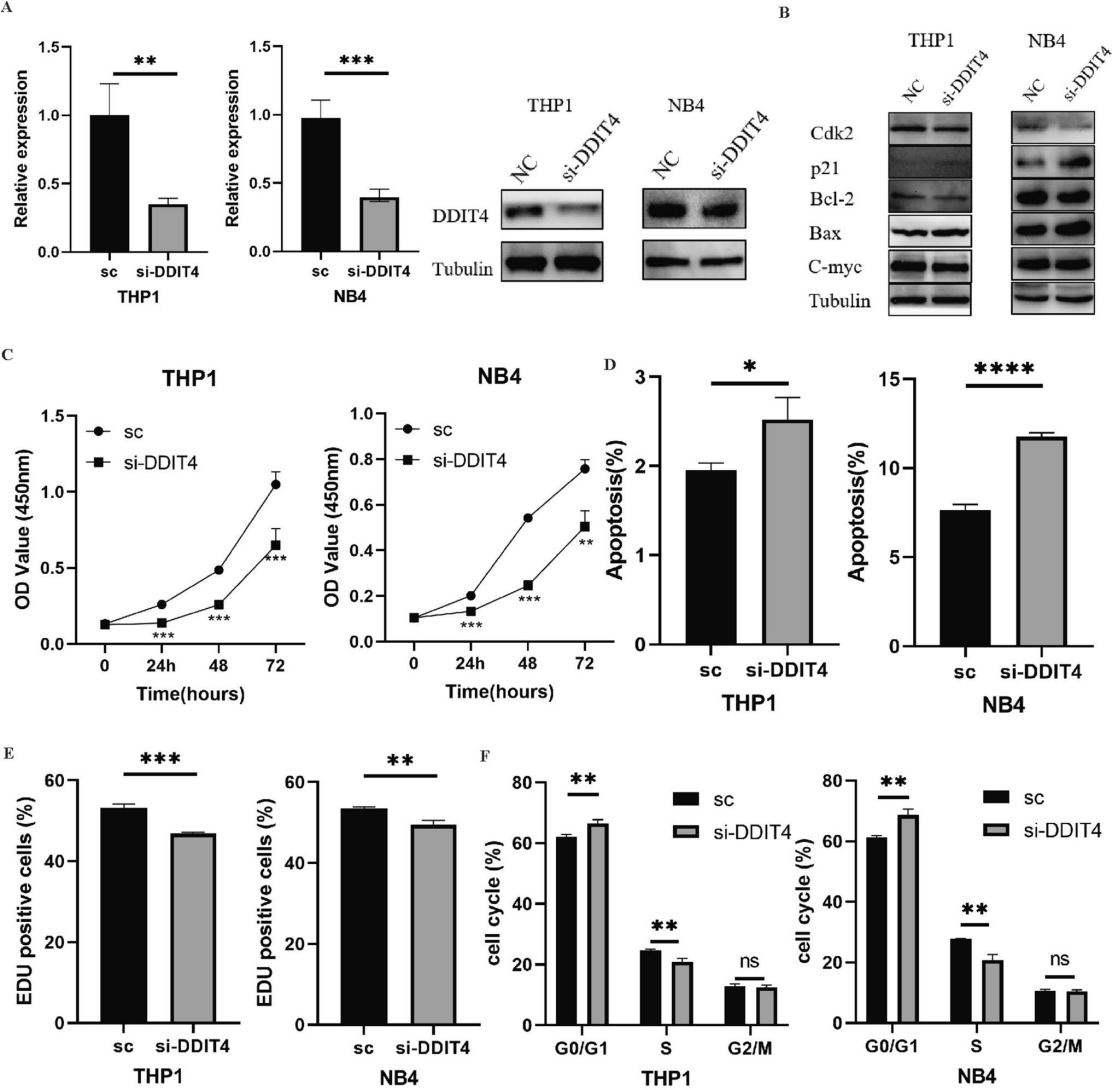
**A** *DDIT4* knockdown efficiency was assessed by qRT-PCR and western blot. **B** Western blot measured apoptosis, proliferation and cell cycle-related proteins. **C** Cell viability was assessed by CCK-8 assay. **D** Apoptosis increased after *DDIT4* knockdown. **E** Cell proliferation ability was tested by EdU assay. **F** The proportion of the G1 stage increased when *DDIT4* was knocked down. (ns *p* ≥ 0.05, *p < 0.05, **p < 0.01, ***p < 0.001)

To investigate the role of *DDIT4* in AML, we used small interfering RNAs (siRNAs) to knock down *DDIT4* in leukemia cell lines THP1 and NB4. The expression of *DDIT4* was significantly decreased (Fig. 7A). The results of CCK-8 showed that knocking down *DDIT4* significantly inhibited the activity of THP1 and NB4 cells (Fig. 7C). EdU proliferation assay confirmed the results that cell proliferation decreased after *DDIT4* knocking down (Fig. 7D). Flow cytometry results showed that after *DDIT4* knockdown, the apoptosis of THP-1 and NB4 cells increased (Fig. 7E), the number of G_0_/G_1_ phase cells increased, and the number of S phase cells decreased (Fig. 7F). Figure 7B showed the expression of apoptosis and cell cycle related-protein of leukemia cells, in which the expression of C-myc, Bcl-2 and Cdk2 were decreased, while the expression of p21 and Bax were increased. Together, these results suggested that *DDIT4* affects the proliferation of AML by influencing cell apoptosis and cell cycle.

### Correlation analysis between *DDIT4* and immune cells and immune checkpoint molecules

**Fig. 8:**
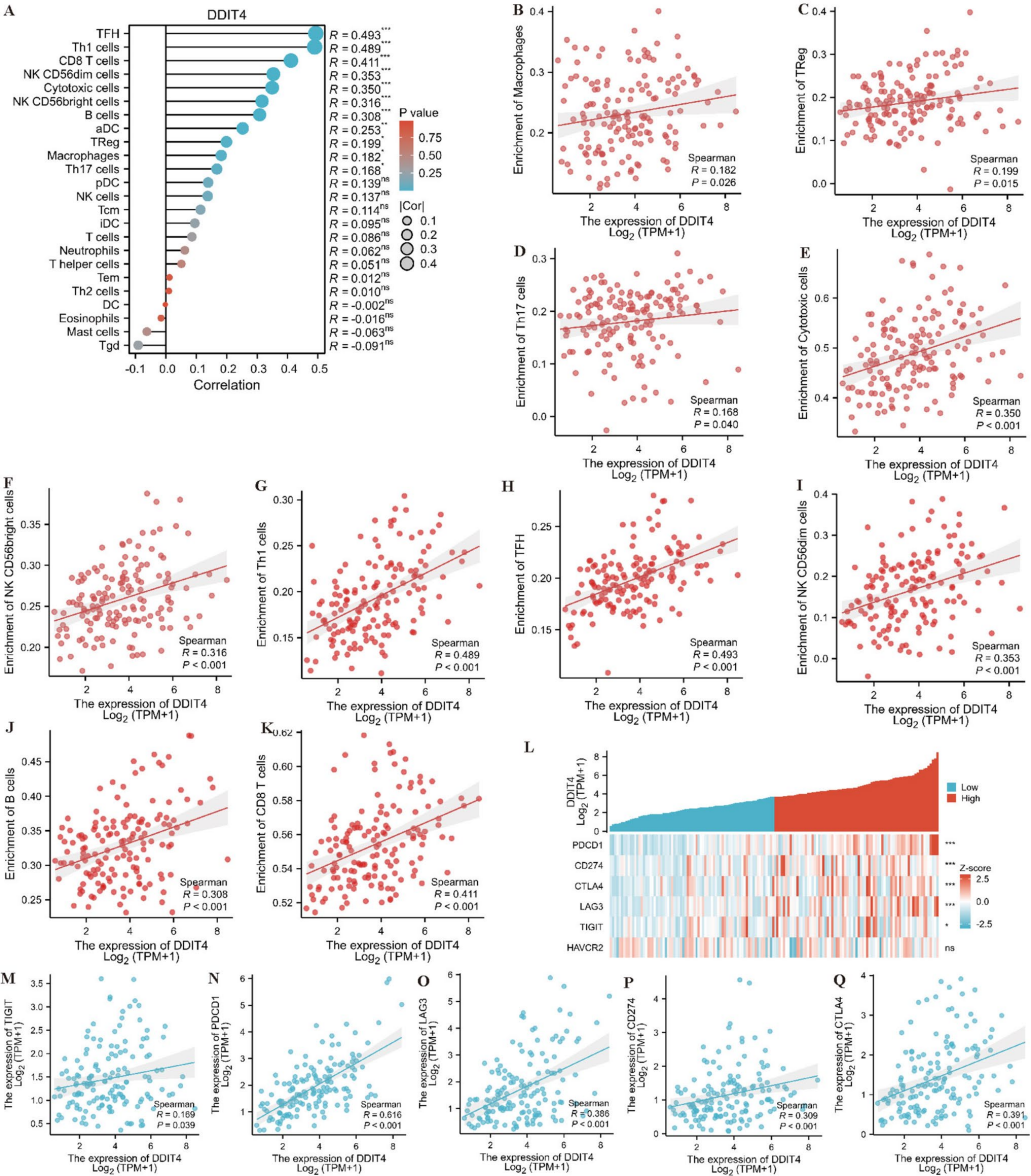
Correlation analysis among *DDIT4* expression and immune cells or immune checkpoint molecules. **A** Relationship among *DDIT4* expression and 24 kinds of immune cells levels. **B**–**K** Correlativity between *DDIT4* expression and immune infiltration in AML. **B** Macrophages. **C** Treg cells. **D** Th17 cells. **E** Cytotoxic cells. **F** NK CD56bright cells. **G** Th1 cells. **H** TFH cells. **I** NK CD56dim cells. **J** B cells. **K** CD8 T cells. **L** Heat map of the relationship between DDIT4 and 6 immune checkpoint molecules (*PDCD1, CD274, CTLA4, LAG3, TIGIT, HAVCR2*). **M**–**Q** Scatter plot of correlation between *DDIT4* and *TIGIT* **M**, *PDCD1* **N**, *LAG3* **O**, *CD274* **P** and *CTLA4* **Q**. (*p < 0.05, **p < 0.01, ***p < 0.001)

To explore the role of *DDIT4* in the immune infiltration of leukemia, we analyzed the relationship between the expression level of *DDIT4* and the expression of 24 kinds of immune cells. Spearman analysis results showed that the expression level of *DDIT4* was positively correlated with Macrophages, Treg cells, Th17 cells, Cytotoxic cells, NK CD56 bright cells, TH1 cells, TFH, NK CD56 dim cells, B cells and CD8 T cells (Fig. 8A). More specific correlation analysis results are shown in Fig. 8B–K. In addition, we also analyzed the relationship between *DDIT4* and 6 classic immune checkpoint genes. As shown in Fig. 8L, the expression level of DDIT4 was positively correlated with the levels of *PDCD1*, *CD274*, *CTLA4*, *LAG3* and *TIGIT*, and the specific analysis was shown in Fig. 8M–Q.

## Discussion

Acute myeloid leukemia is the most common type of acute leukemia in adults. The pathophysiology of AML remains unclear (Stanchina et al. 2020). The available research showed that chromosome aberration, DNA methylation and gene mutation in AML prevented the normal maturation of bone marrow progenitor cells and led to the proliferation of original cell clones (Ley et al. 2013; Nguyen et al. 2019). *DDIT4* plays a driving role in the development of several malignant tumors and it can be induced by a variety of conditions (Pinto et al. 2017). *DDIT4* mainly involves in the PI3K-Akt-mTOR signaling pathway, which regulates cell growth, proliferation and apoptosis (Sofer et al. 2005).

In this study, we used the public database to investigate the mRNA expression and prognostic value of *DDIT4* in pan-carcinoma. We found that *DDIT4* was significantly abnormally expressed in a variety of tumors. *DDIT4* works differently in different cancers. High *DDIT4* expression had better OS in ESCA and worse OS in LUAD, KICH, PAAD, and HNSC. Expression of *DDIT4* in tumors is correlated with cancer type, but high expression is associated with worse staging in most tumors. These results suggest that *DDIT4* expression and prognostic significance were highly cancer-dependent, and the unique role of *DDIT4* in each cancer needs to be further identified. We also studied the clinical significance and effect of *DDIT4* in AML by bioinformatics analysis and functional determination. In our study, *DDIT4* was expressed at a low level in AML patients. Analysis of the clinical features of AML patients in the TCGA cohort indicated that up-regulated expression of *DDIT4* was an independent prognostic biomarker for OS in AML. High expression of *DDIT4* was also associated with unfavorable genetic risk, advanced age (> 60 years) and IDH1-R132 mutations in AML patients. Those results suggest that *DDIT4* may play a role in the pathogenesis of AML.

Chemotherapy is the preferred treatment for acute myeloid leukemia. Relapse due to chemotherapy resistance is the leading cause of death in AML patients (Kantarjian et al. 2021). Studies have shown that drug resistance in tumor cells is partly due to the acquisition of anti-apoptotic capabilities and the failure of DNA damage response and repair (Assaraf et al. 2019). Lin Fu, et al. reported that patients with elevated *DDIT4* expression who received chemotherapy alone had a worse prognosis and might benefit from allo-HSCT (Cheng et al. 2020). To explore the potential mechanism of *DDIT4* affecting the prognosis of AML, patients were divided into two groups with high *DDIT4* expression and low *DDIT4* expression, and the differential genes in these two groups were analyzed by GO and KEGG pathways. The results showed that DEGs were related to the PI3K-Akt signaling pathway and apoptosis signaling pathway. PI3K-Akt is a key pathway that regulates cell growth, proliferation and apoptosis (Porta et al. 2014). We knocked down *DDIT4* in AML cells, and the decrease of *DDIT4* in THP-1 and NB4 cell lines showed increased apoptosis, decreased proliferation, and cell cycle arrest in the G_0_/G_1_ phase, indicating that *DDIT4* has a carcinogenic function in AML.

The expression of *DDIT4* in AML patients is lower than that in healthy controls, but patients with high expression have a poor prognosis, which should involve unknown complex mechanisms. Previous studies have shown that *IL-10* strongly up-regulates *DDIT4* expression and inhibits mTOR activity during macrophage activation (Ip et al. 2017). *IL-6* can reduce *IL-6*-induced mTOR signal activation by decreasing *DDIT4* expression (Pinno et al. 2016). We constructed the PPI network of differential genes and screened the core genes, including *IL-6* and *IL-10. IL-6* is highly expressed in AML and is not correlated with *DDIT4* expression and AML prognosis; *IL-10* is positively correlated with *DDIT4* expression and those with high expression in AML have shorter OS. Core genes *CCL25*, *THBS1*, *MMP7*, *IGF1* and *HGF* were found to have an effect on OS in AML patients. In our current study, we have not found why *DDIT4* expression was reduced in AML, which may involve other mechanisms besides *IL-6* and *IL-10*, which need to be further studied in the future.

Up to now, the outcome of AML is still unsatisfactory, and immune escape of tumor cells plays an important role in the cause of poor response to chemotherapy and recurrence. AML cells can cause changes in the microenvironment of bone marrow tumors to facilitate immune escape (Stanchina et al. 2020). The microenvironment of AML has an inhibitory effect on immune effector cells. Elevated Treg cell-mediated immunosuppression in AML patients allows leukemia cells to evade immune monitoring (Ustun et al. 2011). High levels of macrophage infiltration in tumors are associated with poor prognosis and progression of cancer (Gwak et al. 2015). TH17 cells can increase the expression of Treg cells in the presence of TNF-α, resulting in an immunosuppressive state that promotes the progression of AML (Wang et al. 2018). The role of *DDIT4* in the immune infiltration of AML remains unclear. We analyzed the correlation between *DDIT4* expression and immune infiltration in AML from the data published in the public database, and the results showed that the abundance of Cytotoxic cells, NK CD56 bright cells, TH1 cells, TFH, NK CD56 dim cells, B cells and CD8 T cells significantly increased in the *DDIT4* overexpression group. These results indicated that overexpression of *DDIT4* was associated with increased immune infiltration in AML. The abundance of Macrophages, TH17 cells and Treg cells also increased in the *DDIT4* high expression group. The elevation of these three immune cells should be responsible for the progression of immune escape disease in the group of AML patients with high *DDIT4* expression.

In summary, we found that patients with high DDIT4 expression in AML have a poor prognosis, and *DDIT4* may affect the progression of AML by regulating cell proliferation, apoptosis, cell cycle and immune microenvironment. *DDIT4* is expected to be a potential prognostic marker for AML.

## Data Availability

The orginal data displayed in this study are included in the article. Further requests can be contact to the corresponding author.

## References

[CR1] Assaraf YG, Brozovic A, Gonçalves AC, Jurkovicova D, Linē A, Machuqueiro M, Saponara S, Sarmento-Ribeiro AB, Xavier CPR, Vasconcelos MH (2019) The multi-factorial nature of clinical multidrug resistance in cancer. Drug Resist Updat 46:100645 31585396 10.1016/j.drup.2019.100645

[CR2] Bindea G, Mlecnik B, Tosolini M, Kirilovsky A, Waldner M, Obenauf AC, Angell H, Fredriksen T, Lafontaine L, Berger A, Bruneval P, Fridman WH, Becker C, Pagès F, Speicher MR, Trajanoski Z, Galon J (2013) Spatiotemporal dynamics of intratumoral immune cells reveal the immune landscape in human cancer. Immunity 39:782–795 24138885 10.1016/j.immuni.2013.10.003

[CR3] Cheng Z, Dai Y, Pang Y, Jiao Y, Liu Y, Cui L, Quan L, Qian T, Zeng T, Si C, Huang W, Chen J, Pang Y, Ye X, Shi J, Fu L (2020) Up-regulation of DDIT4 predicts poor prognosis in acute myeloid leukaemia. J Cell Mol Med 24:1067–1075 31755224 10.1111/jcmm.14831 PMC6933361

[CR4] Colwill K, Gräslund S (2011) A roadmap to generate renewable protein binders to the human proteome. Nat Methods 8:551–558 21572409 10.1038/nmeth.1607

[CR5] Döhner H, Weisdorf DJ, Bloomfield CD (2015) Acute myeloid Leukemia. N Engl J Med 373:1136–1152 26376137 10.1056/NEJMra1406184

[CR6] Gwak JM, Jang MH, Kim DI, Seo AN, Park SY (2015) Prognostic value of tumor-associated macrophages according to histologic locations and hormone receptor status in breast cancer. PLoS ONE 10:e0125728 25884955 10.1371/journal.pone.0125728 PMC4401667

[CR7] Hänzelmann S, Castelo R, Guinney J (2013) GSVA: gene set variation analysis for microarray and RNA-seq data. BMC Bioinformatics 14:7 23323831 10.1186/1471-2105-14-7 PMC3618321

[CR8] Ip WE, Hoshi N, Shouval DS, Snapper S, Medzhitov R (2017) Anti-inflammatory effect of IL-10 mediated by metabolic reprogramming of macrophages. Science 356:513–519 28473584 10.1126/science.aal3535 PMC6260791

[CR9] Kantarjian HM, Short NJ, Fathi AT, Marcucci G, Ravandi F, Tallman M, Wang ES, Wei AH (2021) Acute myeloid leukemia: historical perspective and progress in research and therapy over 5 decades. Clin Lymphoma Myeloma Leuk 21:580–597 34176779 10.1016/j.clml.2021.05.016 PMC11938811

[CR10] Ley TJ, Miller C, Ding L, Raphael BJ, Mungall AJ, Robertson A, Hoadley K, Triche TJ, Laird PW, Baty JD, Fulton LL, Fulton R, Heath SE, Kalicki-Veizer J, Kandoth C, Klco JM, Koboldt DC, Kanchi KL, Kulkarni S, Lamprecht TL, Larson DE, Lin L, Lu C, Mclellan MD, Mcmichael JF, Payton J, Schmidt H, Spencer DH, Tomasson MH, Wallis JW, Wartman LD, Watson MA, Welch J, Wendl MC, Ally A, Balasundaram M, Birol I, Butterfield Y, Chiu R, Chu A, Chuah E, Chun HJ, Corbett R, Dhalla N, Guin R, He A, Hirst C, Hirst M, Holt RA, Jones S, Karsan A, Lee D, Li HI, Marra MA, Mayo M, Moore RA, Mungall K, Parker J, Pleasance E, Plettner P, Schein J, Stoll D, Swanson L, Tam A, Thiessen N, Varhol R, Wye N, Zhao Y, Gabriel S, Getz G, Sougnez C, Zou L, Leiserson MD, Vandin F, Wu HT, Applebaum F, Baylin SB, Akbani R, Broom BM, Chen K, Motter TC, Nguyen K, Weinstein JN, Zhang N, Ferguson ML, Adams C, Black A, Bowen J, Gastier-Foster J, Grossman T, Lichtenberg T, Wise L, Davidsen T, Demchok JA, Shaw KR, Sheth M, Sofia HJ, Yang L, Downing JR, Eley G (2013) Genomic and epigenomic landscapes of adult de novo acute myeloid leukemia. N Engl J Med 368:2059–74 23634996 10.1056/NEJMoa1301689 PMC3767041

[CR11] Liu C, Li Y, Wei M, Zhao L, Yu Y, Li G (2019) Identification of a novel glycolysis-related gene signature that can predict the survival of patients with lung adenocarcinoma. Cell Cycle 18:568–579 30727821 10.1080/15384101.2019.1578146 PMC6464579

[CR12] National Cancer Institute. Cancer stat facts: leukemia—acute myeloid leukemia (AML).https://seer.cancer.gov/statfacts/html/amyl.html. https://seer.cancer.gov/statfacts/html/amyl.html. 2021. Cancer Stat Facts: Leukemia—Acute Myeloid Leukemia (AML). https://seer.cancer.gov/statfacts/html/amyl.htmlhttps://seer.cancer.gov/statfacts/html/amyl.html. Accessed 21 Sep 2017.

[CR13] Nguyen CH, Glüxam T, Schlerka A, Bauer K, Grandits AM, Hackl H, Dovey O, Zöchbauer-Müller S, Cooper JL, Vassiliou GS, Stoiber D, Wieser R, Heller G (2019) SOCS2 is part of a highly prognostic 4-gene signature in AML and promotes disease aggressiveness. Sci Rep 9:9139 31235852 10.1038/s41598-019-45579-0 PMC6591510

[CR14] Pinno J, Bongartz H, Klepsch O, Wundrack N, Poli V, Schaper F, Dittrich A (2016) Interleukin-6 influences stress-signalling by reducing the expression of the mTOR-inhibitor REDD1 in a STAT3-dependent manner. Cell Signal 28:907–916 27094713 10.1016/j.cellsig.2016.04.004

[CR15] Pinto JA, Rolfo C, Raez LE, Prado A, Araujo JM, Bravo L, Fajardo W, Morante ZD, Aguilar A, Neciosup SP, Mas LA, Bretel D, Balko JM, Gomez HL (2017) In silico evaluation of DNA damage inducible transcript 4 gene (DDIT4) as prognostic biomarker in several malignancies. Sci Rep 7:1526 28484222 10.1038/s41598-017-01207-3 PMC5431475

[CR16] Porta C, Paglino C, Mosca A (2014) Targeting PI3K/Akt/mTOR Signaling in cancer. Front Oncol 4:64 24782981 10.3389/fonc.2014.00064 PMC3995050

[CR17] Reville PK, Kadia TM (2020) Maintenance therapy in AML. Front Oncol 10:619085 33604298 10.3389/fonc.2020.619085 PMC7884813

[CR18] Shannon P, Markiel A, Ozier O, Baliga NS, Wang JT, Ramage D, Amin N, Schwikowski B, Ideker T (2003) Cytoscape: a software environment for integrated models of biomolecular interaction networks. Genome Res 13:2498–2504 14597658 10.1101/gr.1239303 PMC403769

[CR19] Shimony S, Stahl M, Stone RM (2023) Acute myeloid leukemia: 2023 update on diagnosis, risk-stratification, and management. Am J Hematol 98:502–526 36594187 10.1002/ajh.26822

[CR20] Shoshani T, Faerman A, Mett I, Zelin E, Tenne T, Gorodin S, Moshel Y, Elbaz S, Budanov A, Chajut A, Kalinski H, Kamer I, Rozen A, Mor O, Keshet E, Leshkowitz D, Einat P, Skaliter R, Feinstein E (2002) Identification of a novel hypoxia-inducible factor 1-responsive gene, RTP801, involved in apoptosis. Mol Cell Biol 22:2283–2293 11884613 10.1128/MCB.22.7.2283-2293.2002 PMC133671

[CR21] Small S, Oh TS, Platanias LC (2022) Role of biomarkers in the management of acute myeloid leukemia. Int J Mol Sci 23:14543 36498870 10.3390/ijms232314543 PMC9741257

[CR22] Sofer A, Lei K, Johannessen CM, Ellisen LW (2005) Regulation of mTOR and cell growth in response to energy stress by REDD1. Mol Cell Biol 25:5834–5845 15988001 10.1128/MCB.25.14.5834-5845.2005 PMC1168803

[CR23] Stanchina M, Soong D, Zheng-Lin B, Watts JM, Taylor J (2020) Advances in acute myeloid leukemia: recently approved therapies and drugs in development. Cancers 12:3225 33139625 10.3390/cancers12113225 PMC7692236

[CR24] Ustun C, Miller JS, Munn DH, Weisdorf DJ, Blazar BR (2011) Regulatory T cells in acute myelogenous leukemia: is it time for immunomodulation? Blood 118:5084–5095 21881045 10.1182/blood-2011-07-365817 PMC3217399

[CR25] Vivian J, Rao AA, Nothaft FA, Ketchum C, Armstrong J, Novak A, Pfeil J, Narkizian J, Deran AD, Musselman-Brown A, Schmidt H, Amstutz P, Craft B, Goldman M, Rosenbloom K, Cline M, O’connor B, Hanna M, Birger C, Kent WJ, Patterson DA, Joseph AD, Zhu J, Zaranek S, Getz G, Haussler D, Paten B (2017) Toil enables reproducible, open source, big biomedical data analyses. Nat Biotechnol 35:314–316 28398314 10.1038/nbt.3772 PMC5546205

[CR26] Wang M, Zhang C, Tian T, Zhang T, Wang R, Han F, Zhong C, Hua M, Ma D (2018) Increased regulatory T cells in peripheral blood of acute myeloid leukemia patients rely on tumor necrosis factor (TNF)-α-TNF receptor-2 pathway. Front Immunol 9:1274 29922294 10.3389/fimmu.2018.01274 PMC5996048

[CR27] Zhao X, Li Y, Wu H (2018) A novel scoring system for acute myeloid leukemia risk assessment based on the expression levels of six genes. Int J Mol Med 42:1495–1507 29956722 10.3892/ijmm.2018.3739 PMC6089755

